# Lsh Is Essential for Maintaining Global DNA Methylation Levels in Amphibia and Fish and Interacts Directly with Dnmt1

**DOI:** 10.1155/2015/740637

**Published:** 2015-09-28

**Authors:** Donncha S. Dunican, Sari Pennings, Richard R. Meehan

**Affiliations:** ^1^MRC Human Genetics Unit, MRC IGMM, University of Edinburgh, Western General Hospital, Crewe Road, Edinburgh EH4 2XU, UK; ^2^Centre for Cardiovascular Science, Queen's Medical Research Institute, 47 Little France Crescent, Edinburgh EH16 4TJ, UK

## Abstract

Eukaryotic genomes are methylated at cytosine bases in the context of CpG dinucleotides, a pattern which is maintained through cell division by the DNA methyltransferase Dnmt1. Dramatic methylation losses are observed in plant and mouse cells lacking Lsh (lymphoid specific helicase), predominantly at repetitive sequences and gene promoters. However, the mechanism by which Lsh contributes to the maintenance of DNA methylation is unknown. Here we show that DNA methylation is lost in Lsh depleted frog and fish embryos, both of which exhibit developmental delay. Additionally, we show that both Lsh and Dnmt1 are associated with chromatin and that Lsh knockdown leads to a decreased Dnmt1-chromatin association. Coimmunoprecipitation experiments reveal that Lsh and Dnmt1 are found in the same protein complex, and pulldowns show this interaction is direct. Our data indicate that Lsh is usually diffuse in the nucleus but can be recruited to heterochromatin in a HP1*α*-dependent manner. These data together (a) show that the role of Lsh in DNA methylation is conserved in plants, amphibian, fish, and mice and (b) support a model in which Lsh contributes to Dnmt1 binding to chromatin, explaining how its loss can potentially lead to perturbations in DNA methylation maintenance.

## 1. Introduction

DNA methylation at the 5′ cytosine position (5 mC) in the context of CpG dinucleotides plays a central role in gene repression in most eukaryotes and flowering plants. 5 mC represents 1% of all nucleotides and 4% of all cytosine residues in mammalian genomes and is required for a wide range of biological processes including transcriptional silencing [1]. Additionally, 5 mC is involved in allele-specific genomic imprinting, X chromosome inactivation in female cells, and silencing of retrotransposons in germ cells and the soma [2]. Moreover, embryonic stem (ES) cells lacking DNA methylation are capable of self-renewal but are unable to differentiate [3]. Two classes of DNA methyltransferases have been widely described in animals: Dnmt1 (maintenance methyltransferase) and Dnmt3a/3b (*de novo* methyltransferases) [4–8]. The heritability of the 5 mC mark is, in part, accounted for by the ability of Dnmt1 to faithfully remethylate DNA daughter strands during and after replication ensuring appropriate methylation patterns in future progeny [9]. Considerable efforts have led to the conclusion that the role of the* de novo* methyltransferases is to establish new methylation marks following the postfertilisation wave of demethylation during early embryogenesis and germ cell development [10–12].

Chromatin structure influences transcriptional states within the mouse genome and can be broadly considered in two different flavours: “active” euchromatin which is enriched for histone marks associated with transcriptional activity (i.e., H3K4me3, H3K27ac) and “inactive” heterochromatin (i.e., H3K27me3, H3K9me3) [13–16]. Interestingly, H3K9me3 acts as a ligand for the chromodomain protein HP1*α* thus reinforcing silencing of heterochromatin [17]. Another tier of chromatin conformation is controlled by nucleosome remodelling complexes including the SWI/SNF family [18]. The SNF domain has been shown to be an ATPase-dependent protein domain capable of shifting nucleosomes on chromatin templates, an effect which can expose or obscure transcription factor binding sites [19]. Lymphoid specific helicase (Lsh) (also known as Hells, PASG, and SMARCA6) is a putative member of the SWI/SNF family [20]; in addition to a SNF domain it harbours a helicase motif which can bend/kink DNA and RNA molecules [21, 22]. Taking these two domains together implies that Lsh may be involved in chromatin remodelling. This hypothesis has been supported by the decreased global DNA methylation levels observed at repeat elements and some single-copy genes in Lsh deficient plants and mice [23–25].

Links between Lsh and DNA methylation have been investigated extensively, but the mechanism by which interference of Lsh function contributes to global hypomethylation remains incomplete [26–28]. Lsh was shown to be dispensable for the recruitment of Dnmt1 to normal replication foci during late S phase [29]. However, support for an association between Lsh and the maintenance methyltransferase Dnmt1 was suggested by the finding that transgene silencing mediated via tethered Gal4-Lsh requires Dnmt1 in cooperation with histone deacetylases and the* de novo* methyltransferase Dnmt3b [27]. Evidence of a link between Lsh and heterochromatin structure arose from studies that demonstrated that Lsh association with chromatin is lost in cells treated with the histone deacetylase inhibitor, trichostatin A (TSA), which results in chromatin having a more accessible hyperacetylated signature [29]. Additionally, loss of Lsh leads to the accumulation of the “activating” mark H3K4me2 globally [29], while the repressive mark H3K9me3 is reduced [26]. Lsh knockout mice die perinatally with gross renal defects or shortly after birth with a spectrum of organ defects and a premature aging phenotype [30, 31].

We present evidence that Lsh is essential for the completion of a normal developmental program in amphibian and fish [32]. In addition, we report that Lsh and Dnmt1 can interact directly* in vivo* and* in vitro* but rarely colocalise at heterochromatic foci in cells; however this can be enhanced by the presence of HP1*α*. Finally, we show that both Lsh and Dnmt1 are chromatin bound and that Lsh is required to recruit or facilitate the association between Dnmt1 and chromatin. Taken together, this study demonstrates that Lsh and Dnmt1 are key protein partners and this may underlie the loss of DNA methylation and embryonic defects that occur in Lsh depleted embryos.

## 2. Materials and Methods

### 2.1. Embryos, Morpholinos, TNT Assay, and TUNEL

*Xenopus laevis* and zebrafish were maintained using standard procedures. All morpholinos were designed and obtained from GeneTools LLC.* Xenopus laevis* 2-cell embryos were microinjected into each blastomere (0.5–10 ng per cell) and allowed to develop. Zebrafish stocks were maintained and embryo cultures were as described previously [32]. Morpholinos were injected (5–10 ng per cell) at the 1-cell stage. Morpholino sequences: xLMO (5′-AGCTCTGTCCCACAGGCATCTTATA-3′; 5′-TTGGGTCATCATCAGATGGTTCCAT-3′); zLMO (5′-GCTTGCTTTTTTCCATTGTGGTCTC-3′); control-MO (5′-CCTCTTACCTCAGTTACAATTTATA-3′). TNT assays were performed using a full-length cDNA xLsh clone as template and were labelled with ^35^S-methionine. Assays were performed in the presence or absence of 200 nM morpholino and products were separated by PAGE. TUNEL staining was carried out as described [33]. TNT assays for GST-pulldowns were carried out by amplifying T7 tagged mLsh and mDnmt1 by PCR with linker primers. PCR products were added to the TNT Quick T7 for PCR DNA kit and translated in the presence of ^35^S-methionine. Whole mount* in situ* hybridisation was carried out as previously described [34].

### 2.2. Southern Blotting and DNA Dot Blotting

Genomic DNA was isolated from embryo batches (~50–100) in SETN buffer: 1% SDS, 1 mM EDTA, 10 mM Tris pH8, and 150 mM NaCl. Lysates were RNaseA treated, proteinase K treated, phenol-extracted, and precipitated yielding high-integrity genomic DNA. For southern analysis, 2–4 *μ*g of DNA were digested to completion with 10 U* HpaII* or* MspI* in a reaction volume of 100 *μ*L for two hours at 37°C followed by a further addition of 5 U enzyme overnight at 37°C. Southern blots were carried out using established methods and probes for xSatI [35] and Dana [36]. For dot blots DNA was dotted onto PVDF (Bio-Rad) and membranes were baked for 2 hours at 80°C under vacuum and then probed with a monoclonal anti-5-methylcytosine antibody (Eurogentec).

### 2.3. Bisulfite Sequencing

Genomic DNA (500 ng) was bisulfite converted using EZ DNA Methylation-Lightning Kit (Zymo Research). PCR primers used were xSatI-Bis1 GTTAATATTAATTTGAGGTTTAG; xSatI-Bis2 GTTTGAATAGTTTAGTTGGTAG; xSatI-Bis3 AAATACTAAATAAAAAAACCC; xSatI-Bis4 TTCAAACTAATACTAAACAAAC. PCR products were cloned into pGEM-T Easy (Promega) and sequenced using BigDye 3.1 sequencing chemistry (Thermo Fisher Scientific) on an ABI Prism 3700 DNA Analyzer (Applied Biosystems).

### 2.4. Cell Culture

Mouse 3T3 and N2a cells and human 293T and SW620 cells were grown in DMEM supplemented with 10% serum and 1% penicillin-streptomycin. p53^−/−^ and p53^−/−^/Dnmt1^−/−^ mouse embryonic fibroblasts were grown as described [37].

### 2.5. GST Pulldowns and Immunoprecipitations

GST and GST-fusions were produced in BL21 cells and crudely isolated using BugBuster (Novagen) followed by binding to glutathione beads (GE HealthSciences). For pulldowns from extracts, nuclear-enriched fractions were isolated using an established method [38]. Briefly, cells were washed in PBS and lysed using RSB-150 (10 mM Tris pH 7.5, NaCl 150 mM, 2.5 mM MgCl_2_, 40 *μ*g/mL digitonin, and protease inhibitor tablets (Roche)) on ice for 5 minutes followed by centrifugation at 2000 g for 8 minutes at 4°C. The cytoplasmic supernatant was discarded and the pellet was resuspended in RSB-150 supplemented with 0.5% Triton X-100, passed through a 40 *μ*M needle and harvested as before. Nuclear extract supernatants were mixed with GST proteins for 1 hour at 4°C, washed in RSB-150 three times, resuspended in Laemmli buffer, and separated by PAGE. Tagged proteins were transfected into 293T cells using Lipofectamine-2000 (Invitrogen) and nuclear extracts were prepared as above. Extracts were precleared with protein G (AutoGen Bioclear) and supplemented with antibodies overnight at 4°C. Finally, fresh protein G was added for one hour at 4°C and complexes were washed three times with RSB-150 and separated by PAGE.

### 2.6. Direct Fluorescence

p53^−/−^ MEF cells were grown on coverslips and transfected with the indicated plasmids using Lipofectamine-2000 (Invitrogen) for 24 hours. Cells were washed twice in PBS, permeabilised in 0.1% Triton X-100/PBS and stained in 0.1 mg/mL DAPI. Coverslips were mounted in Vectashield on slides and were visualised using an Axioplan fluorescence microscope (Carl Zeiss, Welwyn, UK) fitted with a Chroma 84000 quadruple-band pass filter set (Chroma Technology, Rockingham, VT). Grayscale images were captured with an Orca AG CCD (Hamamatsu Photonics, Welwyn Garden City, Hertfordshire, UK).

### 2.7. siRNA, Sucrose Gradients, and Micrococcal Nuclease Assays

siRNA duplexes (sequences available on request) against human Lsh were obtained from Ambion (USA) and were introduced into 293T cells using Oligofectamine (Invitrogen). Knockdown efficiency was determined by immunoblotting using a rabbit polyclonal Lsh antibody (gift from Kathrin Muegge) compared to endogenous PCNA (Abcam) levels. Soluble chromatin was released from mouse 3T3 nuclei using micrococcal nuclease (MNase) overnight and fractionated over 6–40% isokinetic sucrose gradients as described [39]. Fractions were precipitated using an equal volume of 20% trichloroacetic acid and protein pellets were washed in cold acetone and prepared for PAGE in Laemmli buffer. DNA was isolated from gradient fractions by ethanol precipitation and separated on 1x TPE agarose gels. MNase release of chromatin associated proteins was essentially performed as described [40]. Briefly, nuclei were isolated from p53^−/−^ cells using RSB-150 containing 0.5% Triton X-100 and 20 *μ*g DNA equivalents of nuclei were equilibrated in 60 *μ*L solution C (300 mM sucrose, 50 mM Tris pH8, 25 mM KCl, 4 mM MgCl_2_, and l mM CaCl_2_). Aliquots were either untreated or digested with various unit amounts of MNase for 15 mins at room temperature and reactions were stopped by supplementing with 20 mM EDTA on ice for a further 15 mins. Released proteins in the supernatant were isolated by centrifugation at 13,000 rpm for 10 mins at 4°C and both soluble and pellet fractions were processed for protein isolation. The pellet fractions were also processed for DNA isolation to monitor the dynamics of chromatin digestion by MNase.

### 2.8. Western Blotting

Western blotting was carried out using standard methods. In brief, proteins were resolved on 4–12% precast gradient gels (Invitrogen) and transferred to PVDF (Bio-Rad). Blots were blocked in 5% marvel milk in PBS supplemented with 0.1% Tween 20 and incubated with the appropriate antibody at 4°C overnight. Western blot signals were detected using alkaline-phosphatase secondary antibodies (Bio-Rad) and exposed to film (GEHealthSciences).

## 3. Results and Discussion

### 3.1. Lsh Is Essential for* Xenopus laevis* and* Danio rerio* Development

**Figure 1:**
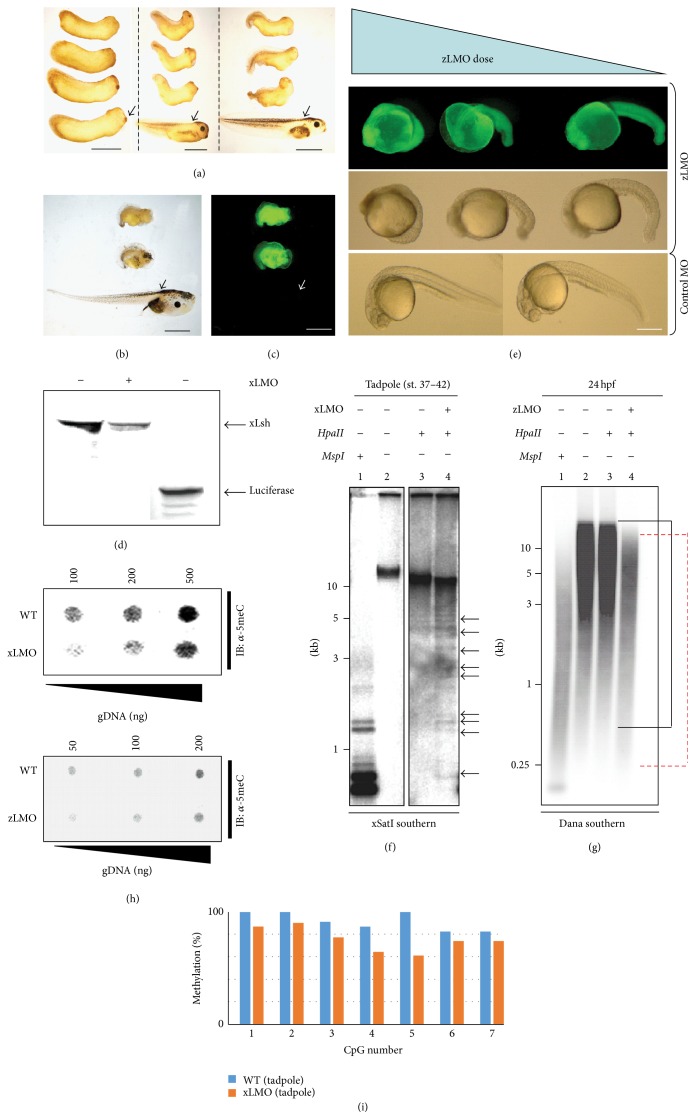
Lsh is essential for both* Xenopus laevis* and* Danio rerio* development. (a–c)* Xenopus laevis* embryos were injected with xLMO or control morpholinos and allowed to develop. Each panel shows examples of morphant embryos and a control embryo (black arrows). xLMO is fluorescein labelled and successfully injected embryos can be visualised under UV light (c). Developmental stages are (a) 28, 37-38, 42, (b) 42–45, (c) 42–45. Scale bar = 1 mm. (d)* In vitro* inhibition of xLsh coupled transcription-translation (TNT) with xLMO. ^35^S-Methionine labelled xLsh protein was prepared by TNT in the presence or absence of xLMO and products separated by PAGE. xLsh production was inhibited by xLMO (compare left and middle lanes). Band on lower right is TNT luciferase protein. (e)* Danio rerio* embryos were injected with zLMO and allowed to develop to the midsomite stage (24 hpf). Severity of phenotype is dose-dependent (compare panels left to right). UV light showing successful microinjection of three doses of zLMO and severity of phenotype (top panel, lateral view). Brightfield view of three doses of zLMO (middle panel, lateral view). Two representative brightfield control morpholino injected embryos (lower panel, lateral view). Scale bar = 300 *μ*m. (f) Southern blot analysis of genomic DNA isolated from control- and xLMO-injected tadpole embryos using a dispersed repeat xSatI probe. DNA was digested with either* HpaII* (methylation-sensitive) or* MspI* (methylation-insensitive* HpaII* isoschizomer), resolved and probed with radiolabelled xSatI. Digestion with* HpaII* indicates that xLMO DNA from tadpoles is more frequently cut as indicated by the low molecular weight banding pattern (black arrows) compared to control-injected genomic DNA. (g) Southern blot analysis of genomic DNA isolated from control- and zLMO-injected 24 hpf embryos using a* Danio rerio* Dana probe. A similar approach was taken as in (f). Compare the extent of* HpaII* digestion in lane 3 (control) and lane 4 (zLMO). Black bracket = wild type* HpaII* profile; dashed red bracket = zLMO* HpaII* profile. DNA sizes are indicated in kilobases to the left of each gel. (h) Upper: dot blot of* Xenopus laevis* genomic DNA probed with 5-methylcytosine antibody. Note the weaker binding of antibody to the xLMO DNA indicating global hypomethylation; lower: dot blot of* Danio rerio* genomic DNA probed with 5-methylcytosine antibody. Note the reduced binding of antibody to the zLMO DNA indicating global hypomethylation. (i) Summary of bisulfite sequencing of xSat in wild type and xLMO tadpole embryos. Vertical axis: % methylation; horizontal axis: each CpG in xSat amplicon.

Lsh orthologs are highly conserved from yeast to humans, and both temporal and spatial analyses show that xLsh is expressed largely ubiquitously throughout all* Xenopus laevis* embryonic stages (see S1-S2 in Supplementary Material available online at http://dx.doi.org/10.1155/2015/740637). Studies in plants and mice have indicated that interference with endogenous Lsh function by gene targeting results in partial hypomethylation of the genome [23, 24, 26]. To address whether this finding is conserved in amphibia and fish we depleted Lsh in* Xenopus laevis* and* Danio rerio* embryos by microinjection with antisense morpholinos that inhibit translation of the target mRNA [41].* Xenopus* Lsh (xLsh) morphants (xLMO) appeared normal through the midblastula transition (MBT) and neurulation. In contrast, at early tailbud stages many xLMO embryos had an aberrant phenotype in comparison with the control morpholino injected siblings (Figure 1(a), left). xLMO midtailbud embryos are axis-truncated and hyperventralised and do not form proper head structures including the eye, cement gland, and brain structures (Figure 1(a), middle panel). xLMO tadpole abnormalities are more pronounced (Figure 1(a), right) and by stages 44-45 (tadpole) many mutants have no tail structure and lack eyes, mouth, and head structures (Figure 1(b)). Successful microinjection and morpholino stability are verified by UV detection of the morpholino fluorescein tag (Figure 1(c)), indicating that the morpholino is stable* in vivo* for over 3 days. In the absence of a suitable antibody against xLsh, we demonstrated xLMO knockdown efficacy by* in vitro* where translation of xLsh mRNA was reduced reproducibly by 70% in the presence of the morpholino (Figure 1(d)). To rule out nonspecific inhibition by xLMO we repeated the same experiment with recombinant radiolabelled luciferase, which was translated efficiently (third lane in Figure 1(d) and data not shown). Finally, we reproduced the similar axis-truncated late-stage phenotype with an xLMO design targeting a different region of the xLsh mRNA (data not shown). xDnmt1- and xKaiso-depleted embryos both show general patterns of apoptosis that is hallmark of their respective phenotypes [33, 42]. In contrast the xLMO morphants showed no significant TUNEL positive staining (Supplementary S3).

We also tested Lsh depletion by morpholino (zLMO) in the model system* Danio rerio*. Embryos were microinjected and allowed to develop to 24 hours after fertilisation (hpf). By titrating the dose of morpholino injected (5–10 ng/embryo), we observed a developmental phenotype compared to wild type embryos (Figure 1(e), compare zLMO and control MO). The morphant phenotype becomes more pronounced: the tail becomes shorter, somite numbers are reduced, and head and brain structures are primitively formed or absent in a dose-dependent manner. Control morpholino injected embryos are shown in Figure 1(e), bottom panel. For more detailed information on embryo phenotypes and survival rates see Supplementary Figures S4-S5.

### 3.2. DNA Hypomethylation Is Conserved in Lsh Depleted Embryos

Interference with Lsh function in plants and mice leads to a global DNA methylation deficit in embryos and cultured cells [43]. Loss of* Arabidopsis thaliana* repeat-associated DNA methylation leads to increased rates of retrotransposition, while loss of repetitive DNA methylation and some single-copy genes occurs in Lsh^−/−^ embryos. Whether this is restricted to plants and mammals is unknown. Previously, we have shown that cytosine methylation is reduced at an interspersed repeat sequence xSatI in xDnmt1-depleted* Xenopus* embryos [42]. Using a similar approach, we tested if DNA hypomethylation occurs at xSatI in xLMO morphants by comparing the digestion profile of genomic DNA using* HpaII* (methyl-sensitive) and* MspI* (methyl-insensitive). In neurula staged embryos, we detected no detectable change in methylation (data not shown). Upon probing with a radiolabelled xSat probe, xLMO tadpole stage (coincident with the morphant phenotype) embryonic DNA is sensitive to* HpaII* digestion compared to control embryonic DNA (Figure 1(f); compare low molecular weight smear in lanes 3 and 4; ethidium gel in Supplementary S6) confirming loss of DNA methylation. We note that this loss of methylation is partial as* HpaII* does not digest to the same extent as* MspI*.

To extend this analysis to fish, we digested control and zLMO genomic DNA isolates from* Danio rerio* as above and probed with a radiolabeled short interspersed repeat element sequence termed Dana [36, 44]. The range of the mean size* HpaII* digested zLMO DNA is shifted compared to the mean size of the control DNA, but we did not observe the appearance of the low molecular weight band observed for* MspI* digestion (Figure 1(g), compare lanes 3 and 4; black bracket (wild type); red bracket (zLMO); ethidium gel in Supplementary S6). This suggests, like Lsh depletion in mouse and* Xenopus*, that loss of DNA methylation in zLMO morphants is partial, consistent with an incomplete knockdown. To validate the observed restriction digestion DNA hypomethylation results, we performed dot-blot analysis using a 5-methylcytosine antibody. Using this approach, we can distinguish between control DNA and xLMO/zLMO DNA, which has approximately 50% less methylated DNA signal compared to the control (Figure 1(h)). Blots were stained with methylene blue to show equal DNA loading (Supplementary S6; [45]). Taken together, these data imply that Lsh is essential for normal development in frogs and fish and that morphant embryos show partial losses in global DNA methylation levels. Finally, we used bisulfite sequencing to examine repeat methylation [34] in xLMO tadpole DNA compared to wild type DNA, showing loss of methylation from the xSat interspersed repeat in morphant DNA across seven CpG positions (Figure 1(i)). Taken together, this suggests evolutionary conservation in Lsh function as a regulator of DNA methylation between plants, fish, frogs, and rodents.

### 3.3. Lsh and Dnmt1 Interact* In Vivo* and* In Vitro*

Dnmt1 is the major DNA cytosine methyltransferase in mammalian cells and has a prominent role in the faithful preservation of DNA methylation patterns in daughter cells after DNA replication. The most striking Lsh target sequences at which DNA methylation is lost are repeat elements, which are both templates for the maintenance (Dnmt1) and* de novo* (Dnmt3a and 3b) methyltransferases in mice. To explain the losses of repeat sequence methylation in Lsh depleted cells we hypothesized that Lsh, which lacks an obvious methyltransferase domain, may be a cofactor for Dnmt1 in maintaining DNA methylation levels at repeat sequence loci (and perhaps genes) and directly participate in their silencing [20].

**Figure 2:**
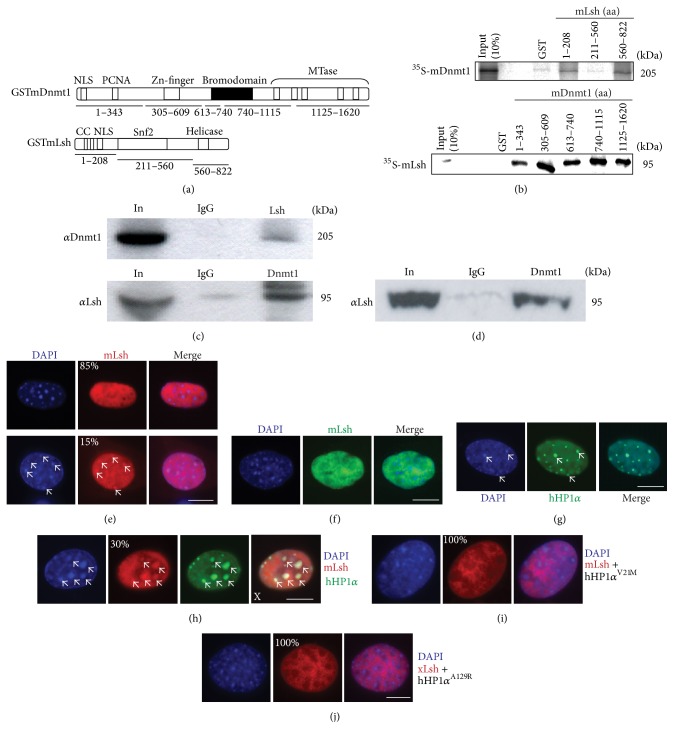
Lsh and Dnmt1 proteins interact* in vitro* and* in vivo* and Lsh is predominantly excluded from pericentric heterochromatin. (a) Cartoon of Lsh and Dnmt1 GST-fusions used. Individual fusions are indicated by numbering under each protein. (b) Direct interaction between Lsh and Dnmt1. Top: mLsh GST-fusions 1–208 and 560–822 pulldown radiolabelled full-length mDnmt1. Bottom: mDnmt1 GST pulldown radiolabelled full-length mLsh. All assays performed in the presence of 50 *μ*g/mL ethidium bromide. (c) Full-length tagged Dnmt1 and Lsh can interact* in vivo* in cultured cells. Tagged proteins (GFP-xDnmt1 and T7-xLsh) were transfected into 293T cells and immunoprecipitated under high salt conditions (250 mM NaCl). Both proteins coimmunoprecipitate reciprocally (see IP lanes, right of each panel). (d) Endogenous immunoprecipitation of human Lsh and Dnmt1 in SW620 cells. (e) Lsh is predominantly nuclear diffuse. Expression of tagged (cherry red) mLsh in p53^−/−^ MEF. White arrows indicate less frequent colocalisation with pericentric heterochromatin. *n* = 100. (f) Expression of previously published [29] GFP-tagged mLsh is nuclear diffuse; in contrast, expression of GFP-tagged HP1*α* overlaps with pericentric heterochromatin foci (white arrows). *n* = 100. (g) Coexpression of Lsh and HP1*α* drives Lsh to heterochromatin. *n* = 80. (h-i) HP1*α* mutants (V21M-chromodomain and A129R-chromoshadow domain) do not redirect Lsh to heterochromatin. *n* = 90.

To test this hypothesis, we carried out biochemical assays to determine if Dnmt1 and Lsh can interact. We first made use of a panel of GST-mLsh and GST-hDnmt1 fusions proteins, which we expressed, purified (Supplementary S6), and used as bait for* in vitro* radiolabeled translated mLsh and mDnmt (Figure 2(a)). We observed GST pulldown signals from the N-terminal and C-terminal domain mLsh GST-fusions for radiolabelled hDnmt1 (Figure 2(b), upper). We note that the N-terminal domain of Lsh contains two coiled-coil domains which are predicted to be protein-protein interaction domains (PFAM: http://hmmer.janelia.org/). Secondly, the C-terminal domain of Lsh encompasses the helicase domain which may imply coupling between Lsh and Dnmt1 at unwinding chromatin. The reciprocal experiment (radiolabeled mLsh and GST-Dnmt1 fusions) showed robust GST pulldown signals for all five hDnmt1 fusions with strongest signals from GST-hDnmt1 (305–609) and GST-hDnmt1 (1000–1632) (Figure 2(b), lower). Next, we tested whether Lsh and Dnmt1 can interact in cellular contexts. We coexpressed full-length tagged Dnmt1 and Lsh fusions in highly transfectable human 293T cells and performed coimmunoprecipitations. Both immunoprecipitated proteins were capable of interacting with the partner tagged protein (Figure 2(c) right, IP lanes). Finally, we wanted to test whether endogenous Lsh and Dnmt1 can interact. Unrelated experiments showed that the human colorectal cancer cell line SW620 expresses high levels of both proteins (data not shown) and blotting of hDnmt1 immunoprecipitates from these cells gave a strong signal using a human Lsh antibody (Figure 2(d)). We also performed these experiments in the high salt conditions previously reported [27] and observed the same interactions (data not shown). Collectively, these biochemical experiments imply that Lsh and Dnmt1 interact* in vitro* and* in vivo* and that this interaction can occur directly without additional nuclear protein partners.

### 3.4. Bulk Lsh Is Predominantly Nuclear Diffuse

Cell biology approaches in cultured murine cells suggest that Dnmt1 is predominantly associated with pericentric heterochromatic nuclear foci at S-phase; however this localisation may fluctuate during the cell cycle and can be lost in cancer cells [46–49]. Others have suggested that Lsh protein expression is essentially nuclear and that this overlaps with Dnmt1 and PCNA at replication foci in late S-phase but not in interphase nuclei [29]. To further explore these findings, we took advantage of a p53^−/−^ MEF cell line [37] which is resistant to overexpression induced cell death to determine Lsh localisation. We observed mouse Lsh (cherry red tagged) to be nuclear diffuse in the majority of nuclei and in some cases present at subtle nuclear foci which overlap in part with pericentric heterochromatin (Figure 2(e), compare upper and lower panels), which implies that the majority of Lsh protein is not associated with pericentric heterochromatin in MEFs. In addition, we tested a T7-tagged xLsh fusion and GFP mLsh in additional mouse cells largely showing diffuse nuclear staining in >90% of cells (Supplementary S7). We detected a similar nuclear diffuse pattern with the previously published GFP-tagged mLsh fusion [29] (Figure 2(f), top panel). In contrast, we observed both GFP-xDnmt1 and GFP-hDnmt1 colocalise with DAPI bright pericentric heterochromatin in up to 50% cells; otherwise these Dnmt1 fusions were nuclear diffuse in the remaining cells (Supplementary S7). Efforts to recruit exogenous Lsh from diffuse nuclear staining to heterochromatic foci in the presence of exogenous Dnmt1 were unsuccessful in p53^−/−^ MEFs; however we observe widespread nuclear diffuse colocalisation implying that these proteins overlap at nonheterochromatic regions in the nucleus (Supplementary S7). In summary, the bulk of Lsh is diffusely stained across nuclei from a variety of cells types with a minor fraction localising with heterochromatic foci.

### 3.5. HP1*α* Can Recruit Lsh to Heterochromatin

A role for Lsh in regulation of histone methylation and the formation of normal heterochromatin was proposed in experiments which demonstrated that H3K4me2 levels were increased in Lsh^−/−^ cells and this could be recapitulated by treating cells with 5′-azacytidine [50]. This suggests a pathway where loss of DNA methylation precedes the gain of activating histone marks at normally silent loci in Lsh^−/−^ cells. Lsh can colocalise with and precipitate HP1*α* after cross-linking suggesting a close (if not direct) association of Lsh with HP1*α* on heterochromatic nucleosomes [29]. Thus, it is possible that HP1*α* facilitates Lsh localisation to heterochromatin. To investigate this we explored the localisation of HP1*α* together with Lsh in p53^−/−^ MEFs. As expected, GFP-HP1*α* localises almost exclusively to heterochromatic DAPI bright spots (Figure 2(f), bottom panel). In the presence of HP1*α* we observed a higher proportion of cells (>30%) exhibiting Lsh accumulation at heterochromatin (Figure 2(g)), compared to expression of Lsh alone (Figure 2(e)) implying that an exogenous pool of active HP1*α* is sufficient to drive Lsh to heterochromatin. Coexpression of HP1*α* mutants with Lsh (HP1*α*V21M: chromodomain mutant; HP1*α*A129R chromo shadow domain mutant) abrogates Lsh presence at heterochromatic foci implying that wild type HP1*α* is sufficient and necessary to recruit Lsh to heterochromatin (Figures 2(h)–2(j)). Interestingly, as we have found for Lsh, HP1 family members are known to interact directly with Dnmt1 and mediate its activity [8].

### 3.6. Lsh Can Recruit Dnmt1 to Chromatin and Can Repress a Nonmethylated Reporter Gene

**Figure 3:**
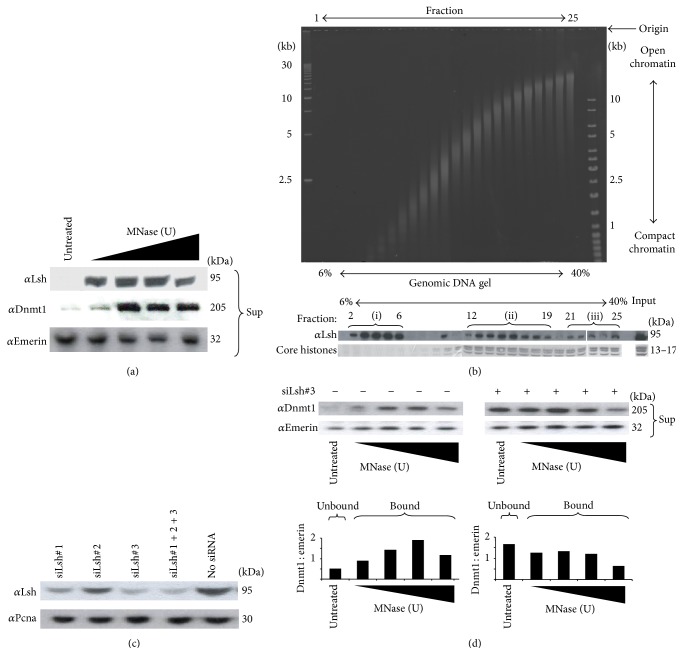
Lsh is associated with chromatin and is required for Dnmt1-chromatin association. (a) MNase treatment of 293T nuclei indicates that endogenous Lsh and Dnmt1 are chromatin bound (see untreated lanes). (b) Endogenous Lsh is associated with soluble chromatin. Sucrose gradient sedimentation was used to fractionate 3T3 soluble chromatin and both protein and genomic DNA were isolated from each fraction. Fractionation of chromatin was validated by DNA gel electrophoresis of all gradient fractions. Western blotting of fractions shows that mLsh (free) is enriched at the top of the gradient (open chromatin) and also cosediments with bulk chromatin (chromatin bound) in the middle and end of the gradient (compact chromatin). (c) siRNAs against human Lsh were tested in knockdown experiments in 293T cells and siLsh#3 gives ~70% knockdown. (d) Lsh is required for the Dnmt1-chromatin association. Comparison of wild type and siRNA treated 293T cells by MNase treatment of nuclei shows that Dnmt1:chromatin association is decreased in knockdown cells (comparison of amounts of Dnmt1 released into the supernatant show higher levels released in knockdown cells). Densitometry of the western blots shows that Dnmt1 is enriched in the chromatin bound fraction (left panel); knockdown of Lsh shifts Dnmt1 into the unbound fraction. Emerin was used as a control for a protein which is unaffected by MNase treatment.

Previous studies have highlighted that Lsh is chromatin associated by showing its presence in the detergent insoluble chromatin fraction derived from mouse nuclei [29]. To test this orthogonally, we examined the coupling of Lsh to chromatin by treating 293T nuclei with micrococcal nuclease (MNase) and assaying for the presence of Lsh in the supernatant (soluble and free) or pellet (insoluble and chromatin bound) [40]. As shown in Figure 3(a), endogenous Lsh is absent from the supernatants of untreated nuclei, in contrast to the high levels present in the soluble fraction of MNase treated nuclei, which demonstrates that Lsh is tightly coupled to chromatin. A similar finding was seen for endogenous Dnmt1 using the same assay (Figure 3(a)). An alternative method of assaying for chromatin bound proteins is fractionating soluble chromatin by sedimentation across sucrose gradients [39] followed by immunoblotting for the protein of interest. We fractionated mouse 3T3 soluble chromatin across isokinetic 6–40% sucrose gradients and precipitated the protein from each fraction and blotted for endogenous Lsh (sedimentation of open and compacted chromatin was confirmed by gel electrophoresis (Figure 3(b))). Three Lsh peaks were observed across the gradient (Figure 3(b): lanes 2–6; lanes 12–19; lanes 21–25) implying that Lsh exists in mouse cells in both monomeric (top of gradient; open chromatin) and in oligomeric nucleosomal fractions (middle (bulk chromatin) and bottom of gradient (compact chromatin)).

Taking the Lsh-chromatin association and Lsh:Dnmt1 interaction data, we tested the hypothesis that Lsh recruits Dnmt1 to chromatin by combining Lsh siRNA knockdown with MNase dependent Dnmt1-chromatin release [40]. Three different siLsh duplexes were transfected into 293T cells (Figure 3(c)) where siLsh#3 achieved highest knockdown of endogenous Lsh levels. In non-siRNA treated cells, Dnmt1 is released after MNase treatment; in contrast Dnmt1 is found in the supernatant of non-MNase treated Lsh knockdown p53^−/−^ cells (Figure 3(d); compare untreated lanes of both top panel western blots). Emerin was used as a control protein, which is not chromatin bound under the conditions used. Densitometry of the western blots was used to calculate a Dnmt1-emerin ratio which illustrates the shift of Dnmt1 from “bound” (no siLsh) to enrichment in the “unbound” (siLsh#3) fraction. These findings suggest the association of Dnmt1 with chromatin can be Lsh dependent.

## 4. Conclusions

A series of investigations have implicated Lsh as a global DNA methylation accessory factor alongside other polypeptides including Dnmt1, Dnmt3a, and Dnmt3b [27, 28]. This role for Lsh was initiated by experiments in DDM1^−/−^ plants (DDM1 is the* Arabidopsis* Lsh orthologue) showing global hypomethylation in these mutants at repeat sequences [24]. This hypothesis was supported when Lsh was knocked out in mice (by two similar strategies) [30, 31] leading to postnatal lethality with concomitant losses in DNA methylation in repeat sequences and more recently at the HoxA gene cluster [51]. This wholesale hypomethylator phenotype in mice was explained by Zhu and colleagues with the finding that Lsh and the* de novo* methyltransferases (Dnmt3a and Dnmt3b) can interact and contribute to the silencing of an episomal transgene independent of DNA replication [28]. We contribute to the current view of Lsh function by reporting that (a) Lsh is essential for frog and fish embryonic development; (b) Lsh and Dnmt1 can associate* in vivo* and interact directly* in vitro*; (c) Lsh recruitment to heterochromatin can be augmented by HP1*α*; (d) the association of Dnmt1 with chromatin is mediated by Lsh.

Interestingly, the phenotype of frog and fish morphants is relatively late-onset (subsequent to the midblastula transition (MBT) and in most cases after neurulation), which is in contrast to phenotypes associated with knockdown experiments of other proteins linked to DNA methylation such as xDnmt1, xKaiso, and xMBD3 [33, 34, 52]. One possibility is that abundant stores of maternal xLsh protein are not depleted by xLMO until later developmental stage (i.e., neurula onwards). An alternative is that Lsh is not essential in early* Xenopus* embryonic genomic silencing. Moreover, we do not see any changes in global DNA methylation until long after the MBT at the tailbud and tadpole stages. The phenotypic effect of Lsh depletion in frogs and zebrafish is not associated with loss of any particular germ layer or organ, which dovetails with the range of phenotypes observed in DDM1^−/−^ and antisense MET1 plants [53, 54]. Similar to what we have established for frogs and fish, in relation to the mouse Lsh phenotype, early development is relatively normal after which mice die either perinatally [30] or a few weeks after birth [31]. These studies report that although embryonic development is overall normal, knockout embryos fail after birth due to a range of defects including renal dysfunction, respiratory problems (lung defects), growth retardation, and an aging phenotype.

In terms of DNA methylation in frog embryo morphants, we observed losses at the high-copy interspersed repeat sequence xSatI. We previously demonstrated that this repeat is heavily methylated in all developmental stages but that this CpG methylation is lost in severely xDnmt1-depleted genomic DNA [42]. The kinetics and extent of xSatI hypomethylation between Lsh and Dnmt1 morphants are different, with partial losses of methylation observed in Lsh tadpole morphants (compared to complete loss at MBT for in Dnmt1 antisense RNA injected mutants). It is possible that Lsh is not involved in maintaining DNA methylation at this repeat in early development but has a more prominent role at late stages. Dnmt1 is highly abundant in early* Xenopus* development and may be sufficient to mediate early repression [34], but as development proceeds its levels are titrated out after multiple cell divisions perhaps permitting Lsh to have a more prominent role in specifying repression at discrete loci.

Here we show a novel direct* in vivo* interaction between Lsh and Dnmt1. Existing data has implied that Lsh interacts predominantly with the* de novo* methyltransferases Dnmt3a and Dnmt3b in MEFs, while this interaction occurs by means of HDAC1 and HDAC2 in transformed cancer cells (HCT116) [27]. Similar to work from Yan et al. [29], we propose that Lsh and Dnmt1 colocalisation in somatic cells is a rare event (<15%). Although this interaction is rare, it is likely to be physiologically relevant as our* in vitro* experiments show a direct interaction between Lsh and Dnmt1 biochemically under physiological salt (~150 mM) conditions and the more stringent conditions (400 mM) employed previously by [27], implying that the interaction is robust even in the presence of ethidium bromide (an inhibitor of DNA:protein interactions). Furthermore, we are able to show immunoprecipitation between Lsh and Dnmt1 in SW620 colorectal cancer cells indicating the proteins are partners* in vivo*. This demonstrates for the first time that while Lsh and Dnmt1 can associate, the* in vivo* protein association may be transient and or cell-cycle regulated. It is a possibility that Lsh cooperates with* de novo* methylation activities in early embryonic cells [28, 55] and that the Lsh and Dnmt1 association is crucial for differentiated and fate-determined soma [27]. Furthermore,* Xenopus* Dnmt3 may not be a* de novo* methylation candidate partner for Lsh as sequence database searches revealed only one Dnmt3 orthologue in the* Xenopus tropicalis* genome that is most similar to murine Dmnt3a2, a truncated form of Dnmt3a lacking the N-terminal 219 amino acids involved in the repression of euchromatic loci [56]. The same homologue is the only Dnmt3-like protein present in the* Xenopus laevis* EST database. Expression analysis of the* Xenopus laevis* transcript indicates that it is only present in later stages of development (Supplementary S8), which argues against* Xenopus* Lsh and Dnmt3a2 having a role in maintaining global DNA methylation during early embryogenesis.

**Figure 4:**
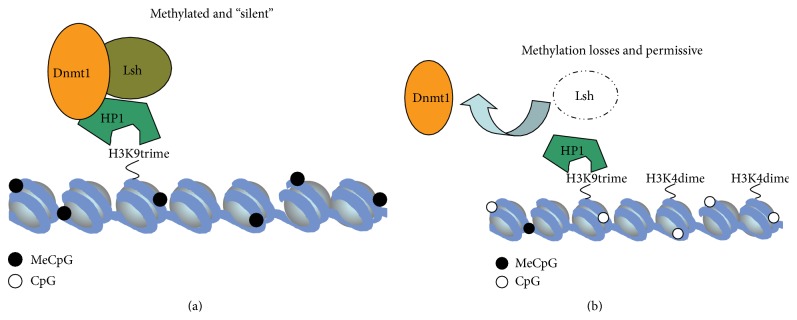
Model for Lsh and Dnmt1 cooperation in silencing. (a) Model for Lsh:Dnmt1 mediated repression. In wild type cells, the H3K9trime mark acts as a ligand in HP1*α* recruitment to silent regions of the genome. Taking together our data and that of others, both Dnmt1 and Lsh can be associated with HP1*α* (perhaps requiring HDACs 1 and 2) thereby allowing the parallel docking of DNA methyltransferase and chromatin remodelling activities to silent loci. (b) In Lsh depleted cells (and knockout plants and animals), targeting of Dnmt1 is diminished leading to reduced DNA methylation maintenance and partial genomic hypomethylation. The accumulation of the activating H3K4me2 mark in Lsh^−/−^ cells may be a downstream effect of DNA hypomethylation.

Nuclear protein localization studies give useful indications of protein function. This is further assisted by the clear staining of blocks of silent pericentric heterochromatin by DAPI (4′,6-diamidino-2-phenylindole) in murine cells which is composed of tandem repeats of satellite sequences. Involvement of Lsh in heterochromatin structure has been reported in mouse Lsh^−/−^ cells which accumulate the activating H3K4me2 mark and by its localisation to DAPI bright spots. In unsynchronised somatic cells (MEFs/3T3/N2a) we rarely (<15%) observe Lsh that is coincident with pericentric heterochromatic foci. Replication of the mammalian genome is organised into early, mid, and late replicating loci with regions containing high gene density early, interspersed repeats later, and condensed heterochromatin at the latest stages of S-phase. Diffuse Lsh staining in >85% of cells may be indicative of localisation at euchromatic gene regions and interspersed repeat sequences. We propose a model where Lsh can cooperate with Dnmt1 at condensed pericentric heterochromatin during late S-phase but these protein partners may also have a role in repressing gene expression (i.e., Hox genes, [51]) and nonheterochromatic interspersed repeat elements and this is facilitated by HP1*α* (see model in Figure 4).

Evidence for a model where Lsh can recruit Dnmt1 to chromatin is strengthened by our MNase release assays which have also been used to demonstrate the association between MeCP2 and chromatin [40]. We show that both Dnmt1 and Lsh are tightly coupled to chromatin in human 293T cells. Using an siRNA strategy to deplete endogenous Lsh we show that the Dnmt1-chromatin association requires normal levels of Lsh. These data are consistent with the idea that Lsh can recruit and modify local nucleosome positioning or act as a cofactor for Dnmt1 binding to chromatin, which would explain the hypomethylation phenotype in Lsh mutants. Interestingly, van Heeringen and colleagues [57] have shown that specific nonmethylated* Xenopus tropicalis* sequences are genetically instructive for H3K27me3 deposition, a finding which supports the opposing paradigm that heterochromatin is epigenetically regulated through recruitment of Dnmt1 to these repetitive genomic regions. Moreover, the action of HDACs may be critical for this process as Lsh-mediated repression of a reporter is alleviated in part by treatment with TSA (data not shown) and the observation that Dnmt1 and Lsh may signal through HDACs [27] (see model in Figure 4). To definitively test these possibilities, sequential ChIP-Seq with antisera against Lsh and Dnmt1 (and Lsh and Dnmt3a/3b) will reveal the genetic targets of these complexes.

## Supplementary Material

Supp S1,2: We initially highlight the conserved nature of the Lsh protein sequence in a range of organisms from lower eukaryotes (yeast) to mammals (mouse and human). This indicates conservation of function has been retained through evolution suggesting the biological importance of Lsh. Moreover, Lsh RNA expression is abundant through all stages of Xenopus laevis, showing high expression in differentiating tissues at later stages.
Supp S3-5: We show , in contrast to xDnmt1-depleted embryos, an absence of apoptotic lineages in xLsh-depleted embryos using TUNEL staining. Next, we provide metrics derived from embryo morpholino injections, which indicate at which stage of development phenotypes arise and the embryonic stages coinciding with lethality.
Supp S6: We provide ethidium bromide stained gels used for Southern blot analysis where it is possible to see DNA hypomethylation in Lsh-depleted genomic DNA. As a control for DNA dotblot analysis, we show a titration of control and xLsh-depleted genomic DNA stained with methylene blue. Next, we provide a coomassie stained polyacrylamide gel showing expression of GST-fusion proteins used in GST-pulldown assays.
Supp S7: This figure provides additional controls and experiments carried out for immunofluorescence experiments.
Supp S8: We show, by RT-PCR, expression of xDnmt3a during the Xenopus leavis developmental stages indicated.
