# Supplementary material for: Lsh Is Essential for Maintaining Global DNA Methylation Levels in Amphibia and Fish and Interacts Directly with Dnmt1
Source: Biomed Res Int. 2015 Sep 28;2015:740637. doi: 10.1155/2015/740637 (PMC4600896; doi:10.1155/2015/740637)
Supplement: Supplementary file 1 — Supp S1,2: We initially highlight the conserved nature of the Lsh protein sequence in a range of organisms from lower eukaryotes (yeast) to mammals (mouse and human). This indicates conservation of function has been retained through evolution suggesting the biological importance of Lsh. Moreover, Lsh RNA expression is abundant through all stages of Xenopus laevis, showing high expression in differentiating tissues at later stages. Supp S3-5: We show , in contrast to xDnmt1-depleted embryos, an absence of apoptotic lineages in xLsh-depleted embryos using TUNEL staining. Next, we provide metrics derived from embryo morpholino injections, which indicate at which stage of development phenotypes arise and the embryonic stages coinciding with lethality. Supp S6: We provide ethidium bromide stained gels used for Southern blot analysis where it is possible to see DNA hypomethylation in Lsh-depleted genomic DNA. As a control for DNA dotblot analysis, we show a titration of control and xLsh-depleted genomic DNA stained with methylene blue. Next, we provide a coomassie stained polyacrylamide gel showing expression of GST-fusion proteins used in GST-pulldown assays. Supp S7: This figure provides additional controls and experiments carried out for immunofluorescence experiments. Supp S8: We show, by RT-PCR, expression of xDnmt3a during the Xenopus leavis developmental stages indicated. [file 740637.f1.zip › 740637.f1/3.pdf]

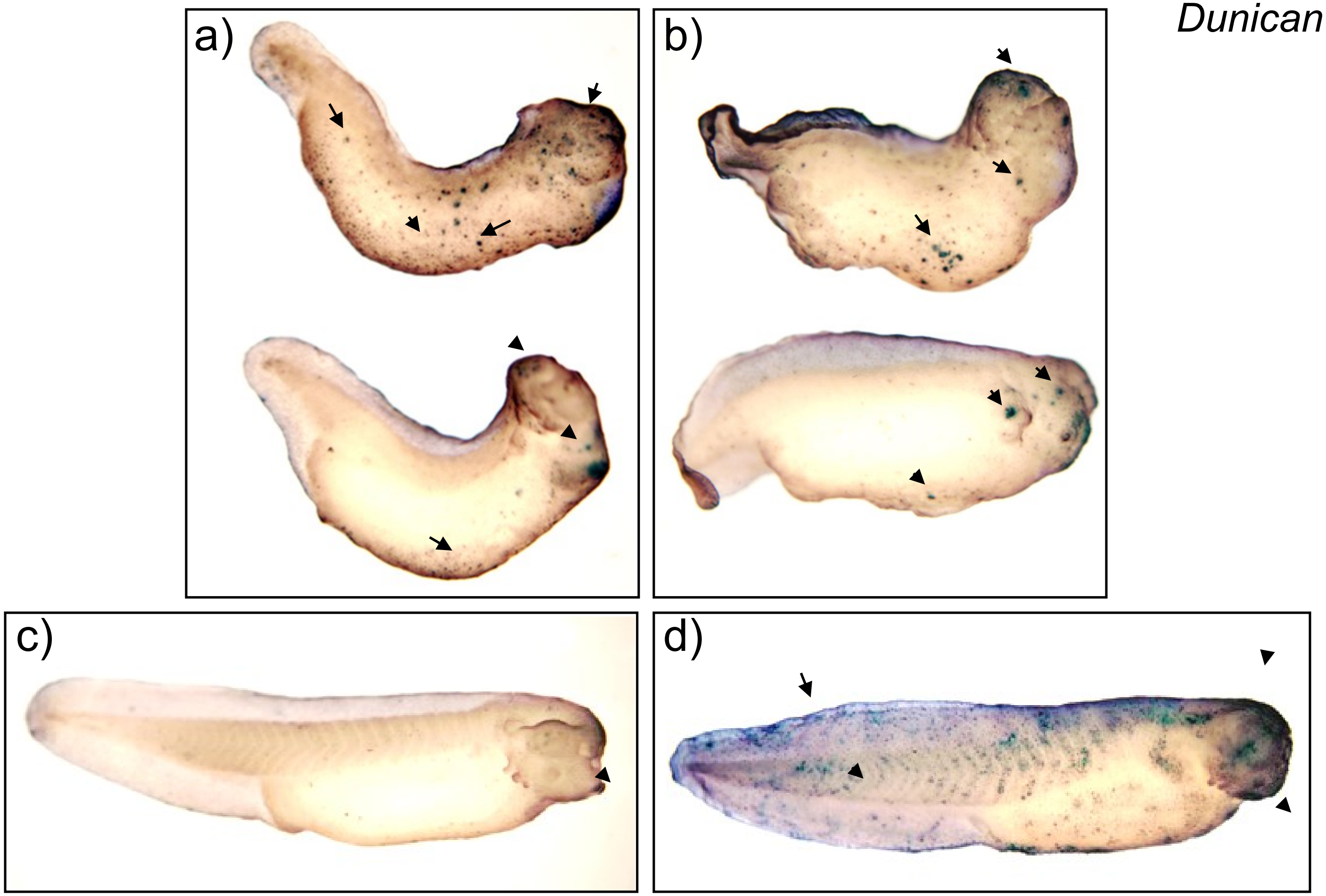

Supp S3 . xLSH morphants are not apoptotic. a) Shown are early tadpole xLMO morphant embryos which show moderate Tunel-positive signals (indicating apoptosis) compared to a wildtype control of the same stage (c) which has no detectable Tunel signals. b) Shown are tadpole xLMO morphant embryos which show moderate Tunel-positive signals (indicating apoptosis) compared to a wildtype control of the same stage (d). Note in (d) the presence of many Tunel positive cells which imply cell lineages undergoing metamorphosis accompanied by cell death. Black arrows indicate Tunel-positive cells. All microinjections are in each blastomere of a 2-cell embryo; morpholino amount = 5-10ng.
