# Supplementary material for: Lsh Is Essential for Maintaining Global DNA Methylation Levels in Amphibia and Fish and Interacts Directly with Dnmt1
Source: Biomed Res Int. 2015 Sep 28;2015:740637. doi: 10.1155/2015/740637 (PMC4600896; doi:10.1155/2015/740637)
Supplement: Supplementary file 1 — Supp S1,2: We initially highlight the conserved nature of the Lsh protein sequence in a range of organisms from lower eukaryotes (yeast) to mammals (mouse and human). This indicates conservation of function has been retained through evolution suggesting the biological importance of Lsh. Moreover, Lsh RNA expression is abundant through all stages of Xenopus laevis, showing high expression in differentiating tissues at later stages. Supp S3-5: We show , in contrast to xDnmt1-depleted embryos, an absence of apoptotic lineages in xLsh-depleted embryos using TUNEL staining. Next, we provide metrics derived from embryo morpholino injections, which indicate at which stage of development phenotypes arise and the embryonic stages coinciding with lethality. Supp S6: We provide ethidium bromide stained gels used for Southern blot analysis where it is possible to see DNA hypomethylation in Lsh-depleted genomic DNA. As a control for DNA dotblot analysis, we show a titration of control and xLsh-depleted genomic DNA stained with methylene blue. Next, we provide a coomassie stained polyacrylamide gel showing expression of GST-fusion proteins used in GST-pulldown assays. Supp S7: This figure provides additional controls and experiments carried out for immunofluorescence experiments. Supp S8: We show, by RT-PCR, expression of xDnmt3a during the Xenopus leavis developmental stages indicated. [file 740637.f1.zip › 740637.f1/7.pdf]

Dunican et al., 2015

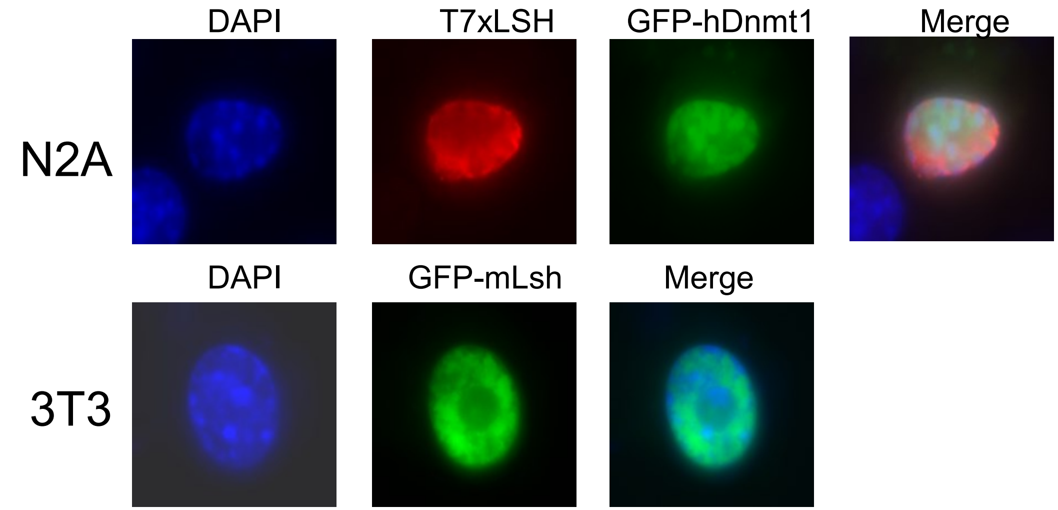

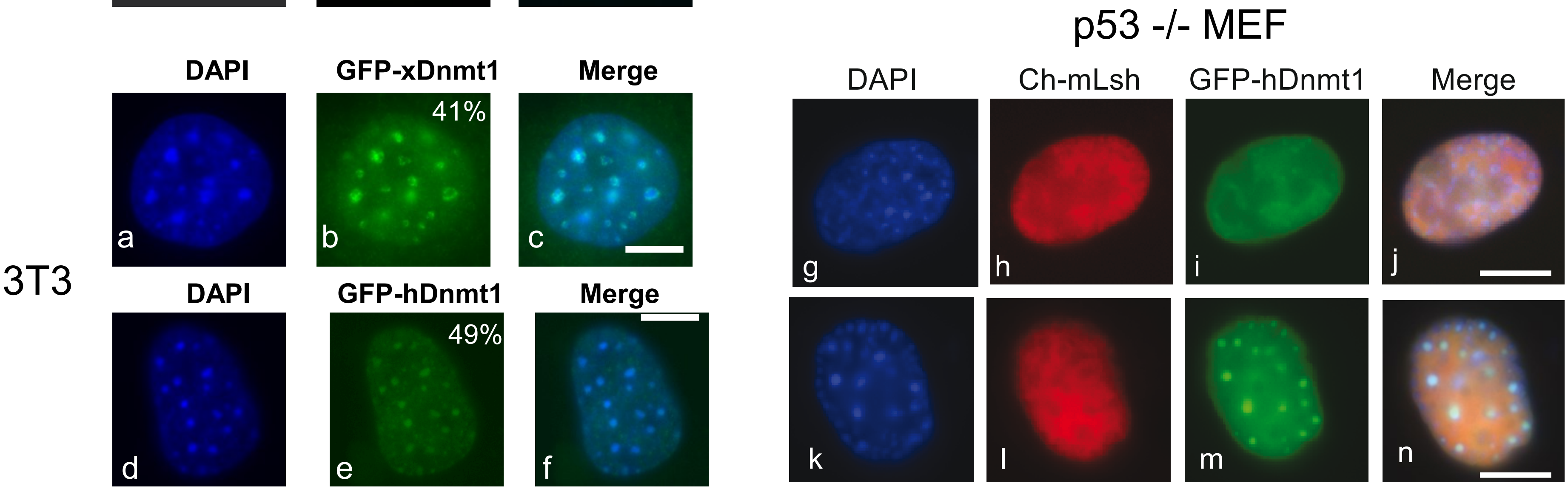

Supp. S7 mLSH is nuclear diffuse in many mouse cell lines. Direct fluorescence of mLSH in two additional mouse cell lines – Neuro2A (N2A) & 3T3. These data show (with the MEF data) that LSH localisation to heterochromatic nuclear foci is a rare event. Additionally, expression of GFP-tagged Xenopus Dnmt1 (xDnmt1) or human Dnmt1 (hDnmt1) in 3T3 cells shows strong association between Dnmt1 and heterochromatic foci (a-f). Lsh and Dnmt1 coexpression does not recruit Lsh to pericentric heterochromatin (g-n)
